# Effect of common horsetail extract on growth characteristics, essential oil yield and chemical compositions of basil (*Ocimum basilicum* L.)

**DOI:** 10.1038/s41598-024-61830-9

**Published:** 2024-05-15

**Authors:** Ghasem Eghlima, Khodabakhsh Goodarzvand Chegini, Mohsen Farzaneh, Fateme Aghamir

**Affiliations:** Department of Agriculture, Medicinal Plants and Drugs Research Institute, Shahid Beheshti University, Tehran, 1983969411 Iran

**Keywords:** Common horsetail, Silicon, Essential oil, Antioxidant activity, GC–MS, Ecology, Evolution

## Abstract

To investigate the effect of horsetail extract containing high silicon on morphological traits, growth, content, and compositions of essential oil of sweet basil (*Ocimum basilicum* L.) an experiment turned into carried out in the shape of a randomized complete block design with three replications. Foliar treatment of horsetail extract with zero, 0.5, 1, and 2% concentrations was applied on 6–8 leaf plants. The assessed traits include plant height, number of leaves per plant, number of sub-branches, leaf area index, plant fresh weight, plant dry weight, total anthocyanin, the content of total phenol and total flavonoid, antioxidant activity, essential oil content, and compounds were measured. The findings demonstrated that the increase of silicon-containing horsetail extract enhanced the improved increase in growth and phytochemical trait values. The use of horsetail extract in the 2% treatment increased plant height, the number of leaves per plant, the number of sub-branches, leaf area index, fresh weight, and dry weight of the plant by 49.79, 45.61, 91.09, 99.78, 52.78 and 109.25%, respectively, compared to the control. The highest content of total phenol (2.12 mg GAE/g DW), total flavonoid (1.73 mg RE/g DW), total anthocyanin (0.83 mg C3G/g DW), and antioxidant activity (184.3 µg/ml) was observed in the 2% extract treatment. The content of essential oil increased with increasing the concentration of horsetail extract, so the highest amount of essential oil was obtained at the concentration of 2%, which increased by 134.78% compared to the control. By using GC–MS, the essential oil was analyzed. The main components of the essential oil include methyl eugenol (12.93–25.93%), eugenol (17.63–27.51%), 1,8-cineole (15.63–20.84%), linalool (8.31–19.63%) and (Z)-caryophyllene (6.02–14.93%). Increasing the concentration of horsetail extract increased the compounds of eugenol, 1,8-cineole, and linalool in essential oil compared to the control, but decreased the compounds of methyl eugenol and (Z)-caryophyllene. Foliar spraying of horsetail extract, which contains high amounts of silicon, as a stimulant and biological fertilizer, can be a beneficial ingredient in increasing the yield and production of medicinal plants, especially in organic essential oil production.

## Introduction

One of the most important medical plants in the Lamiaceae family, basil (*Ocimum basilicum* L.), is used as a fresh vegetable, spice, and medicinal plant^1^. The active ingredients of the vegetative body of this plant have anti-inflammatory, anti-bacterial, anti-fungal, and anti-cancer properties. It is appetizing, and it is used to treat flatulence and help digestion, and it is extensively employed in the sanitary, food, and pharmaceutical industries^2^. Basil contains various biologically active compounds such as phenolic acids, and flavonoids, and the vegetative body of this plant contains between 0.5 and 1.5% essential oil, whose main compounds include methyl chavicol, linalool, camphor, geraniol, and cineole^3^. Plants can serve as the main source of raw materials for synthesizing important and practical biological products, including food, medicine, cosmetics, biopesticides, and biostimulants, because of their high concentration of numerous bioactive substances^4^.

New, effective, and ecologically safe techniques for crop protection, plant growth, and stimulation are needed in the modern period. For these uses, novel, natural, and multi-compound products like plant extracts can be employed. They have anti-fungal, anti-microbial, anti-parasitic, medicinal, antioxidant, aromatic, and anti-inflammatory properties. These natural products can potentially develop into a new biological product generation that may be applied to sustainable agriculture^5,6^. Among higher plants, the aerial sections of horsetails are a substantial source of silicon (Si). Common horsetail (*Equisetum arvense* L.) is an herbaceous, rhizome perennial medicinal plant of the Equisetaceae family and a rich source of silica (above 25% of the dry weight of the plant)^7^. Silicon is one of the nutrients that have multiple roles in the plant, especially under stress conditions and, is considered a multifunctional element in the plant^8^. Some of the most critical roles of silicon in plants include increasing growth, improving yield and quality, improving the photosynthesis process, fixing nitrogen, and creating tolerance against living and non-living stresses^9^. The application of silicon improves the growth and performance traits of *Coriandrum sativum*^10^, *Brassica napus*^11^, *Triticum aestivum*^12^, *Phoenix dactylifera*^13^, and *Echinacea purpurea*^14^. The aim of this research of this have a look at changed into to analyze the impact of different concentrations of horsetail extract (containing high silicon content) on the agro-morphological characteristics, composition, and essential oil content of basil to take steps toward sustainable agriculture by reducing the costs of producing products. It also helped to preserve the environment.

## Materials and methods

### Plant cultivation and experimental design

To investigate the effect of foliar application of horsetail extract on the basil plant, an experiment was conducted in the years 2022 and 2023 in the research farm of the Agricultural Department of the Medicinal Plants and Drug Research Institute of Shahid Beheshti University. This experiment was designed and implemented in the geographical area of 35˚ 48′ N, 51˚ 23′ E, 1776 m altitude in the form of a randomized complete block design with four treatments and three replications. The authors confirm that the necessary permissions to collect and cultivation the samples have been obtained and also the present study complies with the IUCN Policy Statement on Research Involving Species at Risk of Extinction and the Convention on the Trade in Endangered Species of Wild Fauna and Flora. Experimental treatments included control (spraying solution with distilled water), and horsetail extract spraying with concentrations of 0.5, 1 and 2%. For this purpose, after deep plowing of the test plot, disking and complete leveling were done. Before planting the plant, the soil sample was randomly prepared from a depth of 0–30 cm, and some of its physical and chemical characteristics were measured (Table 1). To supply elements required by the plant, before planting seeds, 100 kg per hectare of complete fertilizer was mixed with the soil of the farm. After creating the desired rows, the land was divided into three blocks, each block containing four experimental units, and four piles of 2 m in length were placed in each experimental unit. Basil seeds were obtained from the Pakanbazr Company of Isfahan. After preparing the land, the seeds were sown in early May with 15 cm spacing on the rows and 40 cm between the rows. The plot size was 2 × 2 m, and five rows were planted in each plot. The first irrigation was done immediately after planting the seeds. Necessary crop care, including weeding, was done manually at the necessary times. After the complete establishment of the plant, the first foliar spraying was done at the stage of six to eight leaves, and it was repeated at 15-day intervals until the full flower stage. In general, the plants were sprayed three times during the mentioned period. Morphological and phytochemical parameters were measured in the full flowering stage of the plants.

**Table 1:** Geographical positions, climatic data, and soil properties of the cultivated region.

Geographic positions	Tehran
Longitude (E)	51° 23ʹ
Latitude (N)	35° 48ʹ
Altitude (m)	1776
Climatic data (mean of two years 2022–2023)	
Rainfall (mm)	389
Average annual temperature (ºC)	16.1
Average maximum annual temperature (ºC)	21.5
Average minimum annual temperature (ºC)	11.2
Soil properties	
pH	7.5
Texture	Silty loam
EC (ds m^-1^)	2.93
Total N (%)	0.036
Available P (mg kg^−1^)	58.9
Available K (mg kg^−1^)	338.9

### Preparation of horsetail extract and measuring the silicon content of the extract by ICP-OES

Shoots of *E. arvense* were provided from Mahabad county (36˚ 45′ N, 45˚ 43′ E, 1353 m altitude), located in West Azerbaijan province, at the end of July 2022. Then, they were transferred to the Ecophysiology Laboratory of Shahid Beheshti University (Tehran, Iran), dried at 25 ± 1 ˚C under shade, and kept in a dry and cool place until analysis. The sample was diagnosed in the herbarium of Shahid Beheshti University and registered with the herbarium code MPH-2711. The extract of dry plant samples was prepared by maceration method (extraction with 50% ethanol solvent). For this purpose, the dry powder of the aerial part of the plant was soaked in 50% ethanol for 24 h and shaken. Then, the ethanolic extract was filtered using filter paper and a Buchner funnel. The resulting extract was concentrated and dried by rotary and stored in sterile containers in the dark and the refrigerator until the experiment. For foliar spraying, horsetail dry extracts were prepared in different concentrations of 0.5, 1, and 2%^7^. Silicon content in 0.5, 1, and 2% extracts were 56, 112, and 224 mg/l, respectively. Silicon element was measured with inductively coupled plasma atomic emission spectroscopy (ICP-OES) model 730-ES manufactured by Varian, Australia. The element measurement was done in two steps: first, acid digestion of dry plant samples and then making the necessary standard in the form of a mix. After drying in the oven, the acid digestion of the samples was done well with a concentrated acid mixture containing 65% nitric acid and 35.5% hydrochloric acid within 24 h. Finally, the solutions were heated, filtered, brought to a specific volume, and injected into the ICP device. The element standard was also prepared as a mix for injecting the device. After drawing the standard curve, the final concentration of the element in the samples was obtained^15^.

### Evaluation of Agro-morphological traits and yield

In order to evaluate morphological and functional traits, four subshrubs from each treatment were chosen after the plants had been cultivated for 145 days after the treatment was applied. At the complete blooming stage, morphological characteristics such as plant height, the number of leaves, the number of sub-branches, and the leaf area index were measured. After harvesting, the shoot's clean weight became decided, and then, the plant materials were dried at room temperature. The scale was used to determine the shoot's dry weight after drying.

### Determination of total phenol and flavonoid content

For extracting phenolic and flavonoid compounds, 200 mg of plant samples were powdered. Then, 10 ml of methanol was added to the sample, and placed in an ultrasonic bath for 30 min. Ultrasonic (SingenHtw Elmasonic-D 78224), manufactured by Elma Germany, was placed at room temperature. This step was repeated four times until the maximum amount of phenolic and flavonoid compounds were separated from the plant material and dissolved in methanol. After sonication, the methanolic extract obtained from the four extraction steps was collected and placed in a refrigerated centrifuge model 5702R manufactured by Eppendorf for 10 min at a speed of 4000 rpm. After centrifugation, the upper solution was transferred to dark glasses for the extract and kept in the refrigerator at 4 degrees Celsius until analysis.

To measure the content of total phenol from Power view HT microplate spectrophotometer (Power Wave HT microplate Spectrophotometer (XS2 model, manufactured by BioTeks, USA), and computer data processing system with Gene data analysis software was used. The total content of phenolic compounds in the plant extract was estimated based on Folin's Ciocalteus Reagent (FCR). Accordingly, 25 μl of the tested sample were mixed with 125 μl of diluted FCR solution (1:10), and then 100 μl of 7.5% sodium carbonate solution (Na_2_CO_3_) were added the plate was covered with aluminum foil, and the absorbance of the solutions was read after two hours at a wavelength of 760 nm by an ELISA reader and the number of phenolic compounds of the whole plant equivalent to gallic acid per gram of dry matter was measured^16^.

In this research, aluminum chloride reagent (AlCl_3_) was used to measure flavonoids. For this purpose, 100 μl of distilled water and 7.5 μl of 5% sodium nitrite solution (NaNO_2_) were added to 25 μl of the tested sample. After 6 min of incubation, 7.5 μl of 10% aluminum chloride, 100 μl of 4% sodium hydroxide (NaOH), and 10 μl of distilled water were added to each well. The plate was covered with aluminum foil, and the absorbance of the solutions was read after 15 min at a wavelength of 510 nm by an ELISA reader. The number of flavonoid compounds in the whole plant equivalent to rutin per gram of dry matter was measured^16^.

### Antioxidant activity

The DPPH technique is used by Blois methods^17^ to assess antioxidant activity. In summary, the test tube was filled with 0.2 ml of methanolic extract and 4.0 ml of DPPH solution, and it was then allowed to sit at room temperature for 20 min. A spectrophotometer operating at 517 nm was used to examine the reduction of the DPPH radical. As the control, butylated hydroxytoluene was employed. The IC_50_ values were calculated: DPPH scavenging effect (%) = (Abs_0_ – Abs_1_/Abs_0_) × 100.

$$\begin{aligned} {\text{Abs}}_{0} \, & = \,{\text{The absorbance of the control}} \\ {\text{Abs}}_{{1}} \, & = \,{\text{The absorbance of the sample}}{.} \\ \end{aligned}$$

### Determination of anthocyanin content

For this purpose, 0.2 g of leaves were ground in acidic methanol (including methanol and hydrochloric acid in a ratio of 99:1). The resulting extract was centrifuged for 20 min at 12,000 rpm. The supernatant solution was placed in the dark at laboratory temperature for 12 h, and the absorbance of the samples was read with a spectrometer at a wavelength of 550 nm, for anthocyanin extinction coefficient ε = 3300 Mm^-1^ cm^-1^ and anthocyanin content in μM/g DW was reported^18^.

### Essential oil extraction

Essential oil extraction was done in the full flowering stage using 50 grams of dried aerial parts by water distillation method using a Clevenger-type apparatus. The extraction time for all samples took about 4 hours. The essential oil was collected in separate containers and dehydrated by dry sodium sulfate. The essential oil content was calculated using Eq. 1:

1 $${\text{Essential oil content}}\; \, \left( \% \right) = \left( {{\text{essential oil weight}}/{\text{dry weight}}} \right) \times {1}00$$

To prevent the decomposition of the essential oil by light and heat, the vial containing the essential oil was wrapped in foil and kept in the refrigerator (4 °C).

### Quality and quantity determination of essential oil components by GC and GC–MS

Gas Chromatography (GC) was performed using a Trace GC with a DB-5 column (30 m length × 0.25 mm inner diameter × 0.25 µm film thickness). The oven temperature was programmed to increase from 60 to 250 °C at a ramp rate of 5 °C/min. The injector temperatures were held at 250 °C. Helium gas was used as the carrier gas at a flow rate of 1.1 mL/min, and 1 μl of essential oil samples were injected with a 1:10 split ratio.

Gas Chromatography-Mass Spectrometry (GC–MS): The essential oil samples were analyzed using GC (Trace MS-Thermo Quest-Finnigan) coupled with MS (Quadrupole). The apparatus is equipped with a DB-5 column (30 m length × 0.25 mm inner diameter × 0.25 µm film thickness). The oven temperature was programmed to increase from 60 to 250 °C at a rate of 5 °C/min, and the injector temperatures were held at 250 °C. Helium gas was used as carrier gas with a flow rate 1.1 mL/min. Essential oil samples of 1 μL were injected with a split ratio of 1:10. Ionization energy was set at 70 eV, scan time at 0.4 s, and mass range adjusted between 40 and 460 amu. The essential oil compounds were identified using retention index, mass spectra data, literature, and the National Institute of Standards and Technology (NIST) computer library. Normalizing the surface area and ignoring the response coefficients obtained the relative percentages of essential oil components.

### Statistical analysis

The mean data of both years was used for data analysis. In order to analyze the experimental data, Minitab 16 software was initially used to verify the data's normality. Subsequently, the data were analyzed using the ANOVA function in R 4.0.4 (https://www.r-project.org), and the means were examined using the least significant difference (LSD) test. Excel 2016 was used to design each and every diagram.

## Results

### Morphological and functional traits

Variance analysis of the data revealed that, at a probability level of 5% (*P <* 0.05), the influence of various extract amounts on the morphological and functional features assessed in this experiment was significant. The average height of the plant shows that with the increase in the concentration of the extract, the height of the plant also increases significantly. The highest (57.73 cm) and lowest (38.54 cm) plant height were observed at the level of 2% extract and control, respectively. Increasing the concentration of the extract to 2% increased the height of the plant by 49.79% compared to the control (Table 2). Spraying different levels of the extract on the number of leaves in the plant showed that the application of treatments with different concentrations of horsetail extract increased the number of leaves, so that the highest (89.13) increase was related to the treatment of 2% extract concentration, which caused a 45.61% increase in the number of leaves compared to the control (Table 2). The comparison of the average number of sub-branches in the plant resulting from the effects of different levels of horsetail extract showed that at a concentration of 2%, the highest number of sub-branches in the plant was obtained (18.02), which showed an increase of 91.09% compared to the control (Table 2). The results of applying different concentrations of 0.5, 1, and 2% horsetail extract on the leaf area index showed a significant increase of 30.95, 75.97, and 99.78% of the leaf area index compared to the control, respectively (Table 2). The highest (191.37 cm^2^) and the lowest (95.64 cm^2^) leaf area index were related to the concentration of 2% horsetail extract and control, respectively. The highest (82.41 g/plant) and the lowest (53.94 g/plant) plant fresh weight were obtained under the foliar application of 2% concentration of horsetail extract and the control, respectively (Table 2). Based on the results, it can be concluded that foliar spraying with concentrations of 0.5, 1, and 2% of horsetail extract compared to the control led to an increase of 11.66, 38.33, and 52.78% of plant fresh weight, respectively. The highest dry weight of the plant (17.64 g/plant) was obtained under the treatment of 2% concentration of horsetail extract, which showed an increase of 109.25% compared to the control (Table 2).

**Table 2:** Effect of various concentrations of common horsetail extract on the agro-morphological traits of basil.

Extract concentration (%)	Plant height (cm)	Number of leaves plant^−1^	Number of sub-branches plant^−1^	Leaf area index (cm^2^)	Plant fresh weight (g/plant)	Plant dry weight (g/plant)
0	38.54 ± 2.31^d^	61.21 ± 4.05^c^	9.43 ± 1.03^c^	95.64 ± 1.65^d^	53.94 ± 1.01^d^	8.43 ± 0.23^d^
0.5	43.43 ± 3.54^c^	65.84 ± 3.21^c^	12.81 ± 1.53^bc^	125.25 ± 2.86^c^	60.23 ± 0.82^c^	11.53 ± 0.63^c^
1	51.05 ± 2.53^b^	75.72 ± 2.43^b^	14.43 ± 1.28^b^	168.01 ± 3.01^b^	74.62 ± 2.16^b^	14.71 ± 1.03^b^
2	57.73 ± 4.31^a^	89.13 ± 3.51^a^	18.02 ± 1.83^a^	191.37 ± 4.32^a^	82.41 ± 3.01^a^	17.64 ± 1.41^a^

Data are mean ± standard error (n = 3). Within each column, values with the same letters do not show a significant difference (*P <* 0.05).

### Total phenolic and flavonoid content

**Figure 1:**
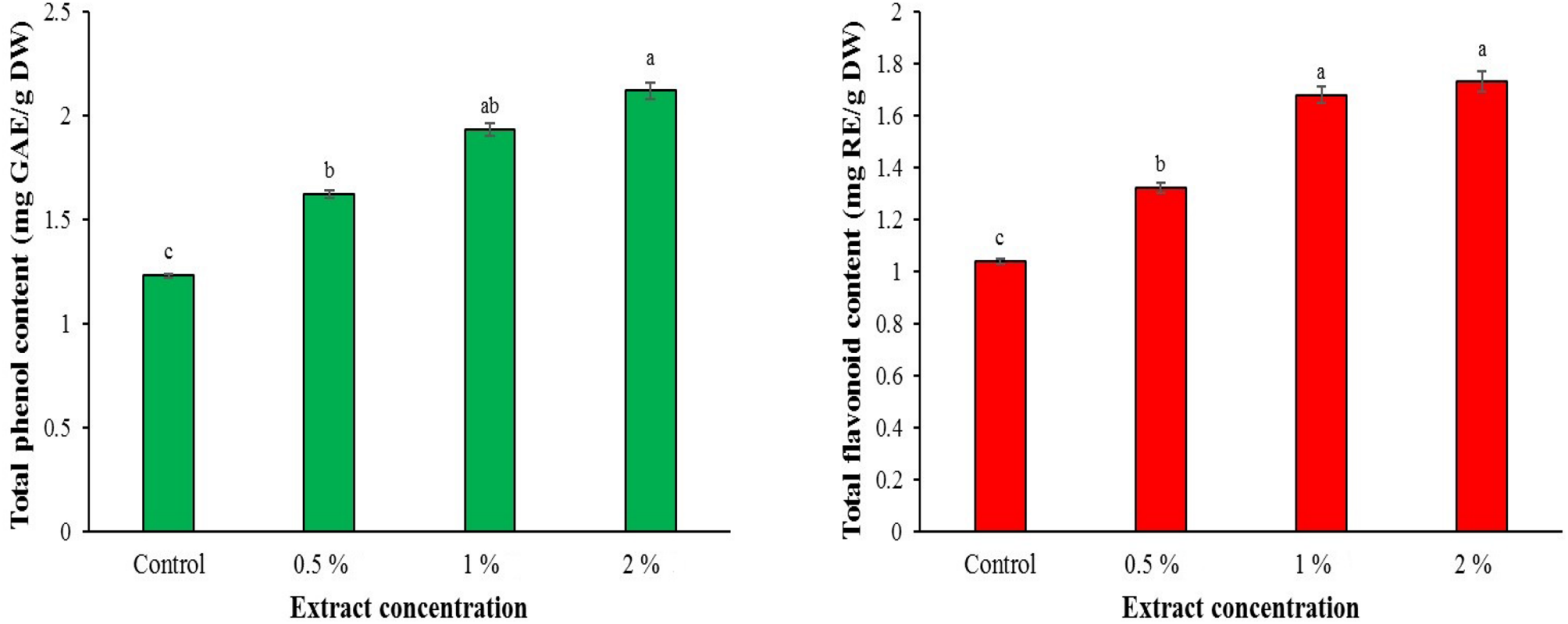
Effect of various common horsetail extract concentrations on the total flavonoid and phenol content of basil.

The results of measuring the content of total phenol and total flavonoid in basil leaves after treatment with horsetail extract showed that the content of total phenol and total flavonoid also increases with the increased in the extract concentration (*P <* 0.05). The highest total phenol content (2.12 mg GAE/g DW) was obtained at a concentration of 2%, which showed an increase of 72.35% compared to the control (Fig. 1). Also, the most (1.73 mg RE/g DW) and the lowest (1.04 mg RE/g DW) total flavonoid content were observed in 2% concentration and control, respectively. Horsetail extract with a concentration of 2% increased the total flavonoid content by 66.34% compared to the control (Fig. 1).

### Total anthocyanin content

**Figure 2:**
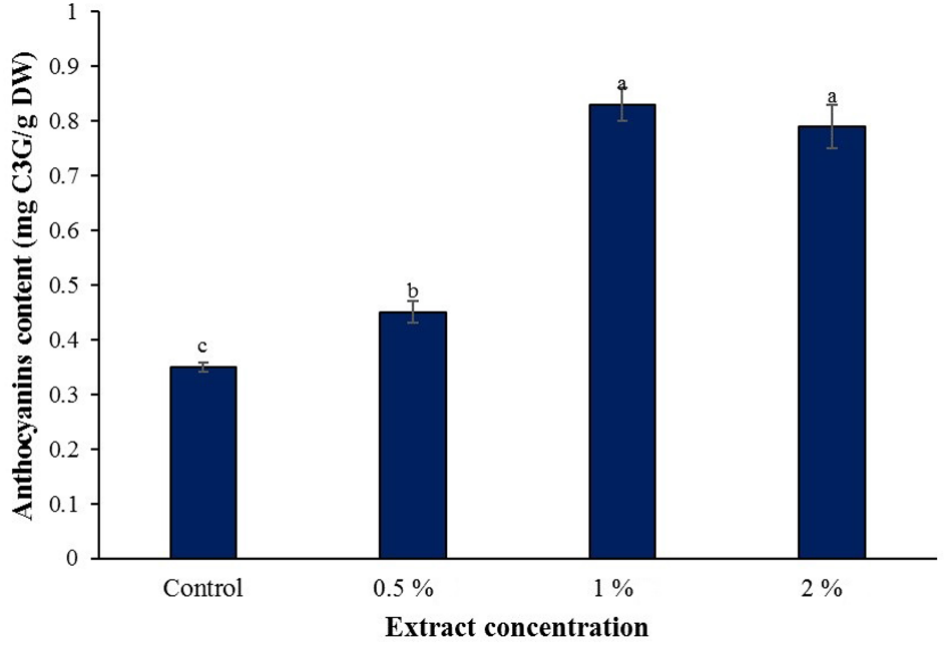
Effect of various common horsetail extract concentrations on anthocyanin content of basil.

Foliar spraying of different concentrations of horsetail extract caused a significant increase in total anthocyanin content in basil leaves (*P <* 0.05). The highest total anthocyanin content of 0.83 mg C3G/g DW and 0.79 mg C3G/g DW was observed in concentrations of 1 and 2%, respectively, which did not have a significant difference with each other. However, it increased the content of total anthocyanin by 137.14 and 125.71%, compared to the control (Fig. 2).

### Antioxidant activity

**Figure 3:**
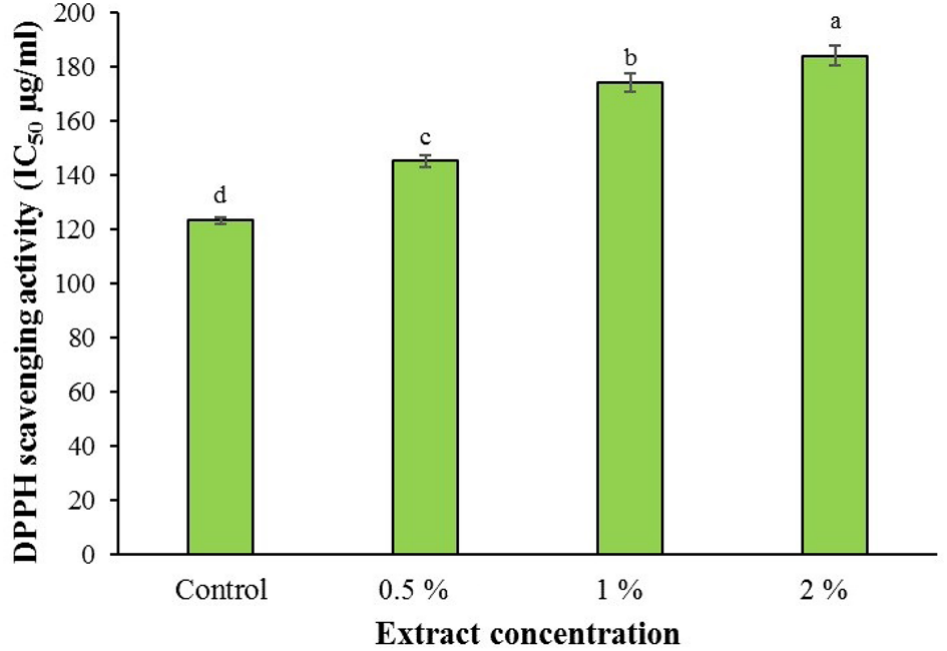
Effect of various concentrations of common horsetail extract on antioxidant activity content of basil.

The capacity of antioxidant activity of the basil plant was influenced by foliar spraying of different concentrations of horsetail extract (*P <* 0.05). Foliar spraying with a concentration of 2% horsetail extract increased the antioxidant activity of basil by 49.35% compared to the control. The amount of antioxidant capacity in 2% concentration of extract and control was 184.3 and 123.4 µg/ml, respectively (Fig. 3).

### Content and composition of essential oils

**Figure 4:**
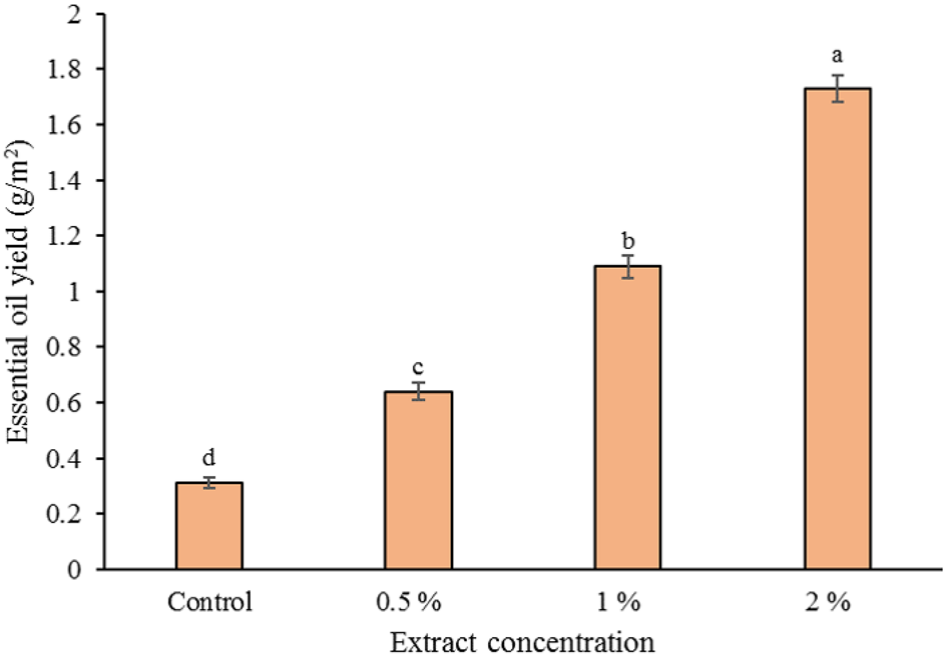
Effect of various concentrations of common horsetail extract essential oil yield of basil.

**Figure 5:**
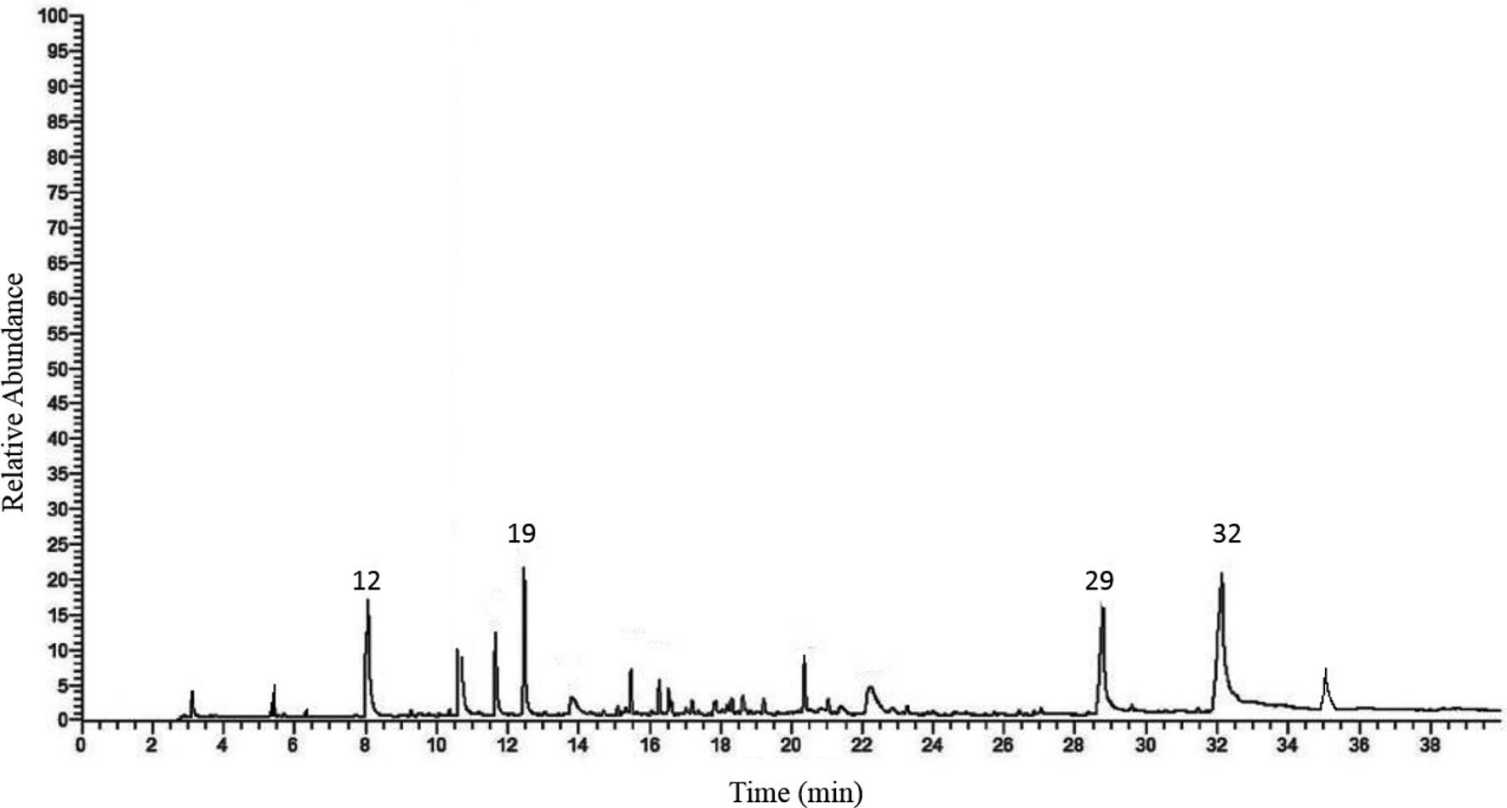
The GC–MS chromatogram of the basil essential oil (the peaks were identified in Table 3, respectively).

This experiment showed that foliar application of horsetail extract had a significant effect on the essential oil content of the basil (*P <* 0.05). All concentrations of horsetail extract used in this study led to an increase in essential oil production in the tested plants. The highest amount of essential oil was found in 2% horsetail extract, which increased by 134.78% compared to the control (Table 3). The essential oil yield was significantly influenced by foliar application of horsetail extract (*P <* 0.05). Increasing the concentration of horsetail extract improved the essential oil yield. The highest yield of essential oil per unit area (1.73 g/m^2^) was obtained by using 2% horsetail extract. The lowest amount was related to the control treatment (0.31 g/m^2^) (Fig. 4). The essential oil of the vegetative body of basil using gas chromatography connected to mass spectrometry (GC–MS) was analyzed and quantified (Fig. 5). The results of GC–MS showed that 35 compounds were detected in the essence of the basil vegetative body treated with horsetail extract. Components of essential oil compounds in Table 3 provided. Based on the results of the main essential oil components in all samples methyl eugenol (12.93–25.93%), eugenol (17.63–27.51%), 1,8-cineole (15.63–20.84%), linalool (8.31–19.63%), and (Z)-caryophyllene (6.02–14.93%) are the main compounds. Treatment with horsetail extract increased the compounds of eugenol, 1,8-cineole, and linalool, while it decreased the amount of methyl eugenol and (Z)-caryophyllene compounds in the essential oil. The highest content of eugenol (27.51%), 1,8-cineole (20.84%) and linalool (19.63%) was obtained in the concentration of 2% horsetail extract, which was significantly different compared to the control (Table 3).

**Table 3:** Effect of different concentrations of common horsetail extract on basil essential oil composition.

No	Composition	RI	Extract concentration (%)			
			Control	0.5	1	2
1	Cumene	901	0.38^b^	0.41^a^	0.27^c^	0.18^d^
2	α-Thujene	903	T	T	T	T
3	α -Pinene	910	1.32^b^	1.53^a^	0.94^c^	0.67^d^
4	Camphene	926	T	T	T	0.21
5	Sabinene	948	1.41^b^	1.63^a^	1.06^c^	0.96^c^
6	*β*-Pinene	953	1.93^d^	2.54^c^	2.74^b^	2.95^a^
7	Myrcene	965	0.52^c^	0.41^d^	0.84^a^	0.62^b^
8	Delta-3-carene	985	T	T	T	0.21^a^
9	α-Terpinene	994	T	T	0.24^b^	0.33^a^
10	*p*-Cymene	1001	T	T	T	T
11	Limonene	1003	0.62^c^	0.66^bc^	0.71^b^	0.80^a^
12	1,8-Cineole	1005	19.54^b^	15.63^d^	17.41^c^	20.84^a^
13	Z-β-ocimene	1007	T	0.13^a^	T	T
14	(E)-b-Ocimene	1012	2.03^b^	2.75^a^	1.61^c^	0.73^d^
15	γ-Terpinene	1018	0.15^b^	0.19^a^	T	T
16	cis-Sabinene hydrate	1024	0.28^c^	0.31^bc^	0.35^b^	0.47^a^
17	Terpinolene	1031	0.14^b^	0.11^b^	0.29^a^	0.31^a^
18	*p*-Cymenene	1034	T	T	T	T
19	Linalool	1038	13.53^c^	8.31^d^	18.07^b^	19.63^a^
20	Camphor	1063	T	T	T	0.17^a^
21	Delta -terpineol	1074	0.41^b^	0.49^a^	0.37^b^	0.32^c^
22	Borneol	1075	T	T	T	0.15^a^
23	Terpinen-4-ol	1079	0.17^b^	0.43^a^	0.15^b^	0.15^b^
24	α -Terpineol	1087	1.53^c^	1.95^b^	1.93^b^	2.12^a^
25	Methyl chavicol (estragole)	1089	0.14^b^	0.23^a^	T	T
26	Octanol acetate	1095	0.21^b^	0.14^c^	0.19^b^	0.32^a^
27	Nerol	1131	T	T	T	T
28	Methyl nerolate	1204	T	0.11^a^	T	T
29	Eugenol	1239	19.76^c^	17.63^d^	24.19^b^	27.51^a^
30	α -Copaene	1260	T	T	0.12^b^	0.21^a^
31	*β*-Elemene	1274	0.31^a^	0.34^a^	0.23^b^	0.14^c^
32	Methyl eugenol	1302	23.51^b^	25.93^a^	19.32^c^	12.93^d^
33	(Z)-Caryophyllene	1324	7.72^b^	14.93^a^	6.98^b^	6.02^c^
34	Germacrene D	1402	T	0.31^a^	T	T
35	Spathulenol	1404	0.10^a^	T	T	0.12^a^
	Monoterpene hydrocarbons		8.50	10.36	8.70	7.97
	Oxygenated monoterpenes		79.08	71.16	81.98	84.61
	Sesquiterpene hydrocarbons		8.03	15.58	7.33	6.37
	Oxygenated sesquiterpenes		0.10	T	T	0.12
	Total		95.71	97.10	98.01	99.07
Essential oil content (%)			0.23	0.34	0.45	0.59

RI: retention indices relative to C6–C25 n-alkanes on the DB-5 column; T: Trace < 0.10%.

## Discussion

Silicon has beneficial effects on the growth and development, morphological traits, and performance of many plant species^9,19^. In previous studies, it has been suggested that the use of silicon increases plant growth by increasing resistance to biotic (pathogens) and abiotic (drought, salinity, heat) stresses^9,20–25^. The importance of silicon in plant nutrition, studies on silicon sources, and their effect on the growth and performance traits of plants are expanding. In foliar application, silicon is absorbed through stomata and deposited in epidermal cells, leading to the formation of hemicellulose in the cell wall^9^. With increasing silicon deposition, the rate of photosynthesis increases and ultimately leads to more biomass production^26^. The use of horsetail extracts improved plant height, number of leaves, number of lateral branches, leaf surface, fresh weight, and dry weight by 49.79%, 89.13%, 91.09%, 99.78%, 52.78%, and 109.25%, respectively, with compared to the control. Studies have shown that silicon supplements affect plant morphology and changes in plant growth characteristics^27,28^. Foliar spraying with silicon in soybean^29^, rice^30^, sorghum^31^, *Kentucky bluegrass*^32^, *Lycopersicum esculentum*^33^, *Lens culinaris*^34^, and *Crataegus aronia*^35^ improved growth traits and biomass. Using silicon through increasing the level of photosynthesis, chlorophyll content, total amino acids, total protein content, nitrogen, phosphorus, and potassium content may increase and improve growth traits as well as increase enzyme activity such as peroxidase and superoxide dismutase enzymes and other processes. This substance is converted into a biochemical that positively affects plant growth increasing the concentration of total phenolic content under biological stimuli, and environmental stresses are considered an adaptive strategy against environmental stresses^36,37^. Increasing the concentration of total phenolic content under biological stimuli and environmental stresses is considered an adaptive strategy against environmental stresses. Phenolic compounds act as antioxidants and produce reduced ROS^38^. In our study, horsetail extract containing silicon increased the content of total phenol, total flavonoid, anthocyanin, and antioxidant activity in basil. In general, different results showed that the accumulation of phenols and increased antioxidant activity increased with silicon^39,40^, which could be caused by the increased expression of the phenylamine lyase gene in the phenylpropanoid pathway^41^. Essential oil components are a group of plant secondary metabolites that are important to both plants and plant consumers. Essential oil content in plants is influenced by genetic factors, environmental conditions, and agricultural techniques^42^. In this research, the content of essential oil in the control, horsetail extract concentration of 0.5%, 1%, and 2% was 0.23%, 0.32%, 0.45%, and 0.59%, respectively, which indicates the increase of essential oil under the effect of foliar application of horsetail extract containing silicon, which is consistent with the results of Farouk et al., that silicon application increases the essential oil content^43^. Silicon plays a vital role in elicitor-accelerating metabolite production by inducing several transcriptional changes^43^. Silicon increases essential oil content by improving cell growth characteristics, ion absorption, density, and size of essential oil glands in leaves^44^. Significant changes in the composition of basil essential oil have been reported in many studies^42,43,45^. In this research, the main components of the essential oil included methyl eugenol, eugenol, 1,8-cineole, linalool, and (Z)-caryophyllene. Based on the results of Chenni et al.^46^, linalool, methyl chavicol, eugenol, methyl cinnamate, 1,8-cineole, bergamotene, limonene, and limonene were the dominant compounds in basil essential oil. The main components of Egyptian basil essential oil have been reported as linalool, 1,8-cineole, eugenol, and methyl cinnamate^47^. Essential oil composition in basil depends on genetic, environmental, and nutritional factors in the plant^42,43^.

## Conclusions

Our results show that foliar spraying of horsetail extract containing silicon as a biological stimulus can increase growth traits and oil content in basil and affect essential oil compounds. As for the increase in the content of phenolic compounds under the influence of different concentrations of the extract, it shows a more efficient antioxidant potential, so the use of basil plant extract and essential oil in the food industry can have a positive effect on quality. Improving food health, so foliar spraying of horsetail extract, which contains high amounts of silicon as a biological stimulus, can be a beneficial substance in increasing the yield and production of medicinal plants, especially in organic essential oil production.

## Data Availability

The datasets generated during and/or analyzed during the current study are available from the corresponding author on reasonable request.
